# Bioactivity-guided isolation of potential antidiarrheal constituents from *Euphorbia hirta* L. and molecular docking evaluation

**DOI:** 10.3389/fvets.2024.1451615

**Published:** 2024-08-29

**Authors:** Junkai Wu, Xiaomeng Zhang, Liyang Guo, Zunlai Sheng

**Affiliations:** ^1^School of Pharmacy, Quanzhou Medical College, Quanzhou, China; ^2^College of Veterinary Medicine, Northeast Agricultural University, Harbin, China; ^3^Heilongjiang Key Laboratory for Animal Disease Control and Pharmaceutical Development, Northeast Agricultural University, Harbin, China

**Keywords:** bioactivity-guided isolation, anti-diarrheal activity, molecular docking, quercitrin, isoquercitrin

## Abstract

**Background:**

*Euphorbia hirta* L., a member of the Euphorbiaceae family, is extensively used as a folk medicine across various regions. In China, its decoction is traditionally consumed to alleviate diarrhea. This study aimed to evaluate the antidiarrheal activities of *Euphorbia hirta* and to identify its bioactive constituents through a bioactivity-guided isolation technique.

**Methods:**

Oral administration of *E. hirta* extract to mice was conducted to assess its effects on diarrhea. The anti-diarrheal effects were investigated in an aqueous extract and in three fractions of varying polarities derived from the aqueous extract, as well as in different eluates from D-101 macroporous resin, and in the compounds quercitrin and isoquercitrin, using mouse models with castor oil-induced diarrhea.

**Results:**

The aqueous extract demonstrated significant anti-diarrheal activities in a dose-dependent manner in the castor oil-induced diarrheal model. Notably, the ethyl acetate (EtOAc) fraction showed prominent effects. Through bioactivity-guided isolation, two major compounds, isoquercitrin and quercitrin from the active fraction were found to possess antidiarrheal effects. Molecular docking studies revealed that the affinity energy of isoquercitrin and quercitrin were −8.5 and −8.2 kcal mol^−1^, respectively, which were comparable to the reference drug loperamide, with an affinity energy of −9.1 kcal mol^−1^.

**Conclusion:**

This research provides evidence supporting the development of *E. hirta* as a therapeutic agent for diarrhea, with isoquercitrin and quercitrin emerging as two key constituents that are likely responsible for its antidiarrheal activity. These findings validate the traditional use of *E. hirta* and highlight its potential as a natural treatment for diarrhea.

## 1 Introduction

Diarrhea is characterized by the alteration of stool consistency to a liquid or mushy state, accompanied by a marked increase in fecal excretion (1). It is a global health issue affecting individuals of all ages, with a disproportionate impact on children under 5 years old (2, 3). Diarrhea is recognized as the second leading cause of mortality in children globally, exerting a significant burden in developing countries (4). The etiology of diarrhea is multifaceted, with intestinal infections and inflammatory bowel diseases being prevalent causes (5–7). Bacterial infections of the intestine can lead to the production of substantial endotoxins, which in turn trigger diarrhea (8). Excessive diarrhea can lead to substantial intestinal tissue damage, underscoring the need for the development of effective anti-diarrheal medications.

Bacterial infections of the intestine can lead to the production of substantial endotoxins, which in turn trigger diarrhea (8). Excessive diarrhea can lead to substantial intestinal tissue damage, underscoring the need for the development of effective anti-diarrheal medications.

*Euphorbia hirta* L., a member of the Euphorbiaceae family, is a small annual herbaceous plant predominantly found in subtropical and tropical regions, including China, Japan, the Philippines, Indonesia, and India (9). It has been utilized in traditional Chinese medicine for centuries to treat gastrointestinal disorders. Phytochemical investigations of *E. hirta* have yielded a diverse array of small molecular compounds, such as coumarin, alkaloids, lignans, flavonoids, terpenoids, and phenols (10–15). These compounds have demonstrated a range of pharmacological properties, including antibacterial activity (16), antiulcer effects (17), anti-inflammatory properties (18, 19), anti-diarrheal capabilities (20, 21), as well as sedative and anxiolytic activities (13, 14, 22). A detailed chemical composition analysis focusing on the phenolic content of *E. hirta* has highlighted the plant’s significant antioxidant and antifungal activities (23, 24). However, the precise mechanism underpinning its antidiarrheal efficacy remains to be fully elucidated.

*Euphorbia hirta* is recognized for its rich flavonoid content, with quercitrin specifically in its antidiarrheal effects (25). Despite this, the presence and contribution of other bioactive constituents within *E. hirta* to its antidiarrheal effects remain elusive, particularly given that most antidiarrheal research to date has focused on complex extracts of the plant. Clarifying these aspects is essential for a comprehensive understanding of its therapeutic potential. Therefore, a bioactivity-guided investigation to screen for active constituents in *E. hirta* is warranted.

The objective of this study was to identify the active constituents of *E. hirta* using a bioassay-guided fractionation approach. We employed liquid extraction, macroporous resin, and silica gel column chromatography to facilitate the preparative separation and purification of bioactive components from the *E. hirta* extract. Subsequently, the interactions between these target compounds and the delta-opioid receptor were assessed using molecular docking. This evaluation aims to inform and support the development of novel antidiarrheal pharmaceuticals.

## 2 Materials and methods

### 2.1 Plant material, reagents and apparatus

#### 2.1.1 Plant material

The entire *E. hirta* plant was collected from Qinzhou City, Guangxi Province, China (N 21°58′47.86′′, E 109°11′22.58′′), during June 2020. Authentication of the plant material was conducted by Professor Huifeng Sun from the Heilongjiang University of Chinese Medicine. A reference specimen (No. V100601) had been deposited in the School of Veterinary Medicine at Northeast Agricultural University, Harbin, China. The plants were air-dried in the shade and subsequently crushed into powder before extraction.

#### 2.1.2 Reagents

Macroporous resin (D101) and silica gel (200–300 mesh) were procured from Nankai Hecheng Science & Technology Co., Ltd. (Tianjin, China). High-performance liquid chromatography (HPLC)-grade methanol and acetic acid were sourced from Merck (Darmstadt, Germany). All other reagents used in this study were of analytical grade and were supplied by Tianjin Yongda Chemical Reagent Co., Ltd. (Tianjin, China). The primers for NHE1, NHE2, NHE3, AQP4, SGLT1, and CFTR were designed and synthesized by Shanghai Shenggong Biology Co., Ltd. (Shanghai, China) (see Table 1).

**Table 1:** Primer sequences used for qRT-PCR.

PCR product	Primer sequences
NHE1	Forward: 5′-TTCATCCACCTCGGATCTTC-3′
	Reverse: 5′-TCTGATGGTGCTGGCAGTAG-3′
NHE2	Forward: 5′-TCATCACGGCTGCTATTGTC-3′
	Reverse: 5′-CACTGACAGCTTGCTGCTTC-3′
NHE3	Forward: 5′-CTATGTGGCTGAGGGAGAGC-3′
	Reverse: 5′-GAGACAGACGCCTCCACAGT-3
AQP4	Forward: 5′-CTGGCCACGCTTATCTTTGT-3′
	Reverse: 5′-CAATGCTGAGTCCAAAGCAA-3′
CFTR	Forward: 5′-CCGGTGACAACATGGAACAC-3′
	Reverse: 5′-AAGAAGCAGCCACCTCAACC-3′
SGLT1	Forward: 5′-AGGCCTGATGCTGTCTGTCA-3′
	Reverse: 5′-CCTTCTTCCGGATCTTGGTG-3′
GADPH	Forward: 5′-CGTGCCGCCTGGAGAAACCTG-3′
	Reverse: 5′-AGAGTGGGAGTTGCTGTTGAAGTCG-3′

#### 2.1.3 Apparatus

All nuclear magnetic resonance (NMR) data for characterising isolated compounds were obtained with the help of Bruker Avance DRX-600 MHz NMR spectrometer (Bruker Corporation, United States), operating at 600 MHz for ^1^H and 125 MHz for ^13^C, respectively. Mass (*m*/*z*) of isolated compounds were determined using Bruker Micro Quadrupole Time-of-Flight (Q-TOF) mass spectrometer (Waters Corporation, United States), equipped with a C_18_ chromatographic column (2.1 mm × 100 mm, 1.7 μm). The melting points of the compounds were determined using a M5000 Automatic Melting Point Apparatus (KRüSS, Hamburg, Germany).

### 2.2 Bioactivity-guided isolation of antidiarrheal constituents from *Euphorbia hirta*

**Figure 1:**
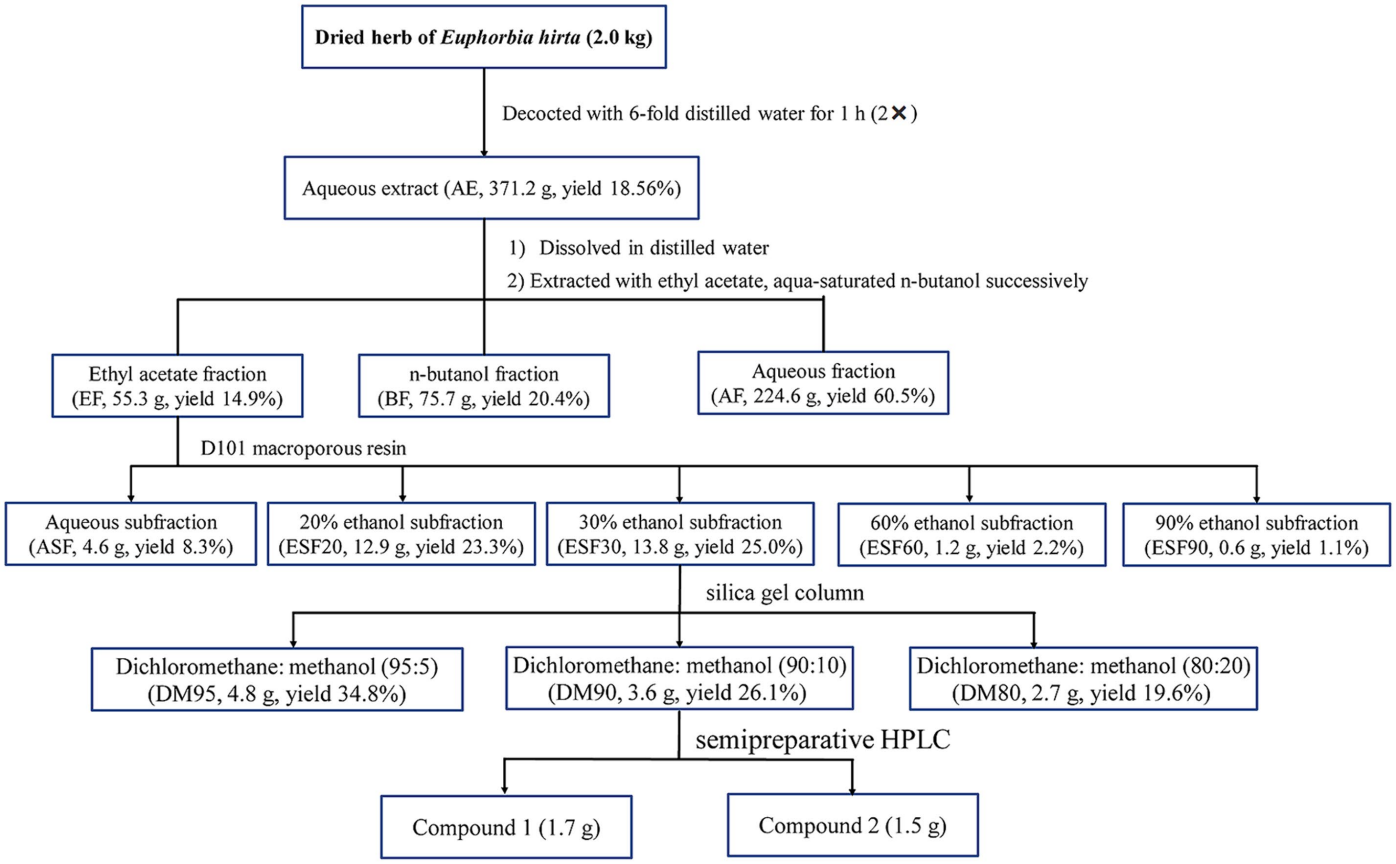
Extraction and fractionation of *Euphorbia hirta* L.

The dried and powdered *E. hirta* (2.0 kg) were decocted two times with a 6-fold volume of distilled water for one hour. The resultant liquid was then subjected to concentration using a RE-52AA rotary evaporator (Shanghai Yarong Biochemistry Instrument Factory, Shanghai, China). Under vacuum conditions at 50°C, the solvent was evaporated, yielding a dried residue of 371.2 g. This residue was suspended in distilled water and sequentially partitioned with ethyl acetate and *n*-butanol, resulting in the isolation of the ethyl acetate fraction (55.3 g), *n*-butanol fraction (BF, 75.7 g) and aqueous fraction (AF, 224.6 g), respectively. The ethyl acetate fraction (EF) was then subjected to column chromatography using D101 macroporous resin, eluting with ethanol of different concentrations to yield five distinct fractions: aqueous subfraction (ASF), 20% ethanol subfraction (ESF20), 30% ethanol subfraction (ESF30), 60% ethanol subfraction (ESF60), and 90% ethanol subfraction (ESF90). Fraction ESF30 (13.8 g) was further separated by silica gel column chromatography, employing a gradient elution system with a mixture of CH_2_Cl_2_-MeOH (95:5, 90:10 and 80:20, v/v), to yield three subfractions: DM95, DM90, and DM80. The combined DM subfraction (3.6 g) was subjected to reversed-phase semipreparative HPLC using a mobile phase of methanol and water (MeOH-H_2_O) to achieve purification and isolation of compounds 1 (1.7 g) and 2 (1.5 g). The detailed procedure for the isolation of the antidiarrheal ingredients (1 and 2) from *E. hirta* is depicted in Figure 1.

### 2.3 Identification of antidiarrheal constituents

Isolated compounds were dissolved in deuterated dimethyl sulfoxide-d6 (DMSO-d6), and their NMR spectra were acquired using a Bruker Avance DRX-600 MHz NMR spectrometer. The carbon and proton chemical shifts were obtained from ^13^C NMR and ^1^H NMR spectra with tetramethylsilane (TMS) as an internal standard.

### 2.4 Pharmacological procedures

#### 2.4.1 Animals and ethics

Kunming mice (20 ± 2.0 g), half male and female, were obtained from the Experimental Animal Center of Harbin Medical University (Harbin, China). Before the initiation of the experimental protocol, the mice were acclimated to a standardized housing environment with a controlled temperature of 20 ± 2°C, relative humidity maintained at 52 ± 5%, and a 12-h light/dark cycle. Food and water were provided *ad libitum* to allow the animals to acclimatize for an initial period of 5 days. All experimental procedures were conducted in accordance with the Guidelines for the Care and Use of Laboratory Animals (Publication No. 85-23, revised in 1985) and were approved by the Animal Ethics Committee of Northeast Agricultural University (No. SRM-08). Every effort was made to ensure the welfare of the animals and to minimize suffering throughout the experimental process.

#### 2.4.2 Preparation of test samples and dose estimation for bioassays

The decoction of *E. hirta* has been traditionally used to treat bacillary dysentery and infectious diarrhea, with a typical ethnomedical dosage of 60 g of crude herb per day for humans (9). In return, the aqueous extract in our experiment was administered orally to the test subjects at dosages of 1,448, 2,895, and 5,791 mg kg^−1^. The doses for the fractions and subfractions were calculated based on their respective yields from the separation process. To evaluate the antidiarrheal effects of the active constituents, isoquercitrin and quercitrin were administered orally at dosages of 12.5, 25, and 50 mg kg^−1^. The negative control group was established by orally administering saline at a volume of 10 mL kg^−1^. Loperamide was used as a positive control, administrated at a dose of 2 mg kg^−1^ (26). All test samples were prepared as suspensions in a 0.5% sodium carboxymethyl cellulose (CMC-Na) solution for the bioactivity assays.

#### 2.4.3 Castor oil-induced diarrhea assay in mice

The antidiarrheal activities of the aqueous extract, fractions and purified compounds were evaluated using a modified protocol based on the method described by Ali et al. (21). Briefly, six mice in each group were subjected to an 18-h fasting period prior to oral administration of the respective samples. After an hour, each mouse was administered castor oil at a dose of 10 mL kg^−1^ to induce diarrhea. The animals were individually housed in cages with white blotting paper lining to facilitate observation. They were monitored continuously for a period of 4 h to assess the onset of diarrhea, the mean of wet faeces, and the inhibition rates, which served as parameters for evaluating the anti-diarrheal activity.

#### 2.4.4 Effects of isoquercitrin and quercitrin on small intestinal propulsion

The impact of isoquercitrin and quercitrin on gastrointestinal motility in mice was assessed utilizing the charcoal method (27). Mice were divided into groups of six and fasted for 18 h prior to the experiment. They were then administered the test samples orally. Group 1 served as the control and received saline at a volume of 10 mL kg^−1^. Groups II to VII were pretreated with isoquercitrin and quercitrin at escalating doses of 12.5, 25, and 50 mg kg^−1^, respectively. Group VII was pretreated with loperamide at a dose of 2 mg kg^−1^. Twenty minutes post-administration, each mouse received an oral dose of 0.2 mL of activated charcoal solution. After a subsequent 30-min interval, the mice were humanely euthanized by cervical dislocation. The small intestine was meticulously excised from the pylorus to the cecum, and the length of the charcoal meal progression was measured to evaluate intestinal propulsion.

#### 2.4.5 Effects of isoquercitrin and quercitrin on castor oil induced enteropooling

Following a method previously described by Rudra et al. (28), 48 rats were subjected to an 18-h fasting period prior to being evenly divided into eight experimental groups: negative control group (I), quercitrin treatment groups (II, III, IV), isoquercitrin treatment groups (V, VI, VII), and positive control group (Group VIII). Group I was orally given distilled water at a dose of 10 mL kg^−1^. Groups II to VII were orally administered quercitrin and isoquercitrin at doses of 12.5, 25, and 50 mg kg^−1^, respectively. Group VIII was treated with loperamide at a dose of 2 mg kg^−1^. These treatments were administered 1 h before the oral administration of 10 mL kg^−1^ of castor oil to induce diarrhea. Thirty minutes post-castor oil administration, all rats were humanely euthanized, and samples of the small intestine were collected. The intestinal contents were gently extruded and transferred to a measuring cylinder to record the volume. The inhibitory effect on intestinal propulsion was calculated using the following formula:


Inhibition(%)=(C−T)/T×100

where *C* represents the volume of the intestinal contents in the negative control group, and *T* represents the volume in the treatment groups.

#### 2.4.6 Acute toxicity testing of isoquercitrin and quercitrin

Thirty healthy Kunming mice, with an average weight of 20 ± 2.0 g, were randomly assigned to six experimental groups, each consisting of 10 animals. The mice in each group were administered isoquercitrin and quercitrin orally at dosages of 1, 2, and 5 g kg^−1^, respectively. Throughout the 14-day experimental period, the mortality rate in each group was recorded.

### 2.5 Molecular docking analysis

We conducted molecular docking simulations utilizing the Autodock VINA software. The methodology for protein-ligand preparation and the subsequent docking process is detailed as follows:

#### 2.5.1 Protein preparation

The X-ray crystal structure of the delta-opioid receptor, with the Protein Data Bank (PDB) identifier 4EJ4, was obtained from the Research Collaboratory for Structural Bioinformatics (RCSB) database. Utilizing PyMOL software,^1^ we removed water and ligands from the protein structure. Subsequently, hydrogen atoms were added to the protein, and Gasteiger were computed using AutoDock Tools 1.5.6 software. The protein structure was then converted into the PDBQT format, which is required for molecular docking simulations.

#### 2.5.2 Ligand preparation

The 3D molecular structures of loperamide, isoquercitrin, and quercitrin were sourced from the PubChem database. Following their retrieval, these structures were converted into the mol2 format, a standard for molecular modeling, using the Open Babel software suite. Subsequently, they were further processed and converted into the PDBQT format, which is optimized for molecular docking studies, employing AutoDock Tools 1.5.6 software.

#### 2.5.3 Docking validation

Utilizing the original ligand structures and their intermolecular interactions as a foundation, we constructed a docking grid box for each target protein using PyMOL software combined with the GetBox plugin. This approach facilitated the creation of a defined spatial region for molecular docking simulations. Subsequently, the ligand molecules exhibiting the lowest binding energies within their respective docking conformations were selected for semi-flexible docking studies. The resulting docked complexes were analyzed using the PLIP web tool (PLIP—Welcome^2^) and the efficacy of the docking simulations was assessed based on the calculated affinity values.

#### 2.5.4 Ligand-based pharmacokinetics and toxicity measurement

We employed the online predictive tool admetSAR^3^ to evaluate the pharmacokinetic profiles of the three compounds, including their human blood-brain barrier penetration (HBD), human blood-albumin binding (HBA), logarithm of the *n*-octanol/water partition coefficient (LogP), human intestinal absorption (HIA), hepatic organic anion binding (HOB), and plasma protein binding (PPB). Additionally, the tool facilitated the assessment of toxicological endpoints, such as the probability of carcinogenicity, the likelihood of mutagenicity in the AMES test, and acute oral toxicity. Furthermore, the drug-like properties of isoquercitrin and quercitrin were assessed based on the 5-rule of Lipinski (M.W. < 500; H-bond donors <5; H-bond acceptors <10; Lipophilicity <5 and molar refractivity 40–130) (29).

### 2.6 Statistical analysis

Data are expressed as the mean ± standard deviation (SD). The statistical differences between groups were conducted to assess statistical differences, employing one-way analysis of variance (ANOVA), followed by Tukey’s test. A *p*-value of less than 0.05 was considered statistically significant. All statistical analyses were performed using SPSS software 18.0.

## 3 Results and discussion

### 3.1 Bioactivity-guided separation and purification of *Euphorbia hirta*

As depicted in Table 2, the aqueous extract of *E. hirta* demonstrated a significant reduction in the quantity of wet fecal matter (*p* < 0.05) and a delayed onset of diarrhea (*p* < 0.05) in a dose-dependent response. These findings corroborate the traditional use of *E. hirta* as an anti-diarrheal remedy in folk medicine. Consistent with our results, Hore et al. (20) documented that the aqueous extract of *E. hirta* leaves mitigated the effects of castor oil-induced diarrhea in murine models. However, the specific active constituents responsible for this effect were not elaborated upon in their study. A 70% methanol extract of the entire *E. hirta* plant exhibited anti-diarrheal properties, with quercetin identified as one of its bioactive components (21). Given the widespread acceptance and use of the aqueous extract of *E. hirta* for the treatment of diarrhea among the Chinese population, elucidating its active constituents is both necessary and of significant interest.

**Table 2:** Effects of aqueous extract and different polarity fractions from *E. hirta* on castor oil-induced diarrhea in mice.

Group	Dose (mg kg^−1^)	Mean of wet faeces	Onset of diarrhea (min)	Inhibition (%)
Negative control	10	13.7 ± 2.62	73.2 ± 12.45	—
AE	1,448	8.4 ± 3.12^*^	108.2 ± 21.8^*^	38.7
AE	2,895	7.5 ± 2.54^*^	120.5 ± 13.67^**^	45.2
AE	5,791	5.0 ± 2.36^**^	143.3 ± 11.53^**^	63.5
EF	215	8.9 ± 3.12^*^	95.1 ± 11.72^*^	35
EF	430	8.1 ± 2.54^*^	121.4 ± 13.25^**^	40.9
EF	860	5.3 ± 2.36^**^	153.2 ± 18.75^**^	61.3
BF	295	11.6 ± 1.72	76.6 ± 6.85	15.3
BF	590	10.5 ± 3.62	75.1 ± 8.77	23.4
BF	1,180	10 ± 2.83	86.5 ± 8.98^*^	27
AF	875	11.8 ± 1.96	68.1 ± 7.96	13.9
AF	3,500	11.5 ± 3.55	72.3 ± 6.63	16.1

Values were expressed as mean ± SD (*n* = 6), ^*^p < 0.05 and ^**^p < 0.01 when compared with control group.

The antidiarrheal effects of the fractions derived from the aqueous extract of *E. hirta* are detailed in Table 2. The EF demonstrated a significant therapeutic advantage over the control group, notably in reducing the incidence of wet fecal matter and delaying the onset of diarrhea (*p* < 0.05, *p* < 0.01). In contrast, neither the n-butanol fraction (BF) nor the aqueous fraction (AF) exhibited discernible inhibitory effects (*p* > 0.05). These findings affirm that the EF is the principal bioactive fraction responsible for the observed antidiarrheal properties of *E. hirta*.

Subsequent fractionation of the EF using macroporous resin D101 yielded five distinct fractions, which were then evaluated in a diarrhea mouse model. As presented in Table 3, the ESF30 significantly mitigated castor oil-induced diarrhea (*p* < 0.05), while the remaining four subfractions did not show significant effects (*p* > 0.05).

**Table 3:** Effects of different subfractions from EF on castor oil-induced diarrhea in mice.

Group	Dose (mg kg^−1^)	Mean of wet faeces	Onset of diarrhea (min)	Inhibition (%)
Negative control	10	13.1 ± 4.79	63.8 ± 7.15	—
ASF	18	11.6 ± 2.12	69.6 ± 9.12	11.5
ASF	36	10.7 ± 3.12	75.9 ± 6.56	18.3
ASF	72	10.4 ± 2.85	75.3 ± 7.01	20.6
ESF20	50	12.3 ± 3.54	70.1 ± 5.84	6.1
ESF20	100	11.8 ± 1.87	78.6 ± 9.74	9.9
ESF20	200	10.5 ± 4.12	77.5 ± 10.91	19.8
ESF30	50	10.5 ± 2.12	82.1 ± 5.42^*^	11.5
ESF30	100	8.4 ± 3.12^*^	116.5 ± 8.76^**^	35.9
ESF30	200	6.4 ± 2.85^**^	138.2 ± 9.88^**^	51.1
ESF60	5	12.5 ± 2.62	68.1 ± 5.92	4.6
ESF60	10	11.3 ± 2.97	76.3 ± 7.67	13.7
ESF60	20	10.5 ± 5.83	87.0 ± 9.41^*^	19.8
ESF90	2	10.8 ± 3.56	68.3 ± 7.32	17.6
ESF90	4	11.6 ± 3.41	70.2 ± 7.41	11.5
ESF90	8	10.9 ± 3.35	65.8 ± 6.81	16.8

Values were expressed as mean ± SEM (*n* = 6), ^*^p < 0.01 and ^**^p < 0.001 vs. negative control group.

The ESF30 was further processed through silica gel column chromatography, eluted with a mixture of dichloromethane and methanol, resulting in three fractions: DM95, DM90, and DM80. Among these, DM90 was identified as a fraction with significant antidiarrheal activity, as documented in Table 4. Finally, the ESF30 was subjected to semi-preparative HPLC using a mobile phase of acetonitrile and water (55:45), leading to the isolation of two compounds with potential antidiarrheal activity.

**Table 4:** Effects of DM95, DM90 and DM80 from ESF30 on castor oil-induced diarrhea in mice.

Group	Dose (mg kg^−1^)	Mean of wet faeces	Onset of diarrhea (min)	Inhibition (%)
Negative control	10	11.1 ± 3.69	73.7 ± 2.49	—
DM95	25	10.6 ± 2.32	77.3 ± 7.83	4.5
DM95	50	9.7 ± 4.23	76.2 ± 5.63	12.6
DM95	100	8.4 ± 2.85	83.5 ± 8.43	24.3
DM90	25	8.3 ± 3.54	80.6 ± 4.57	25.2
DM90	50	7.3 ± 1.87^*^	103.5 ± 7.62^**^	34.2
DM90	100	6.9 ± 4.12^**^	119.2 ± 8.31^**^	37.8
DM80	25	10.5 ± 4.21	75.2 ± 5.31	5.4
DM80	50	9.4 ± 4.22	80.1 ± 3.15	15.3
DM80	100	8.4 ± 3.85	84.4 ± 5.21	24.3

Values were expressed as mean ± SEM (*n* = 6), ^*^p < 0.01 and ^**^p < 0.001 vs. negative control group.

The castor oil-induced diarrhea model has been traditionally used to study diarrhea and evaluate the efficacy of antidiarrheal treatments. However, the castor oil diarrhea model, used to study antidiarrheal treatments, may not accurately represent bacterial diarrhea due to differences in mechanisms, lack of pathogen interaction, and insufficient replication of inflammation and immune responses. Therefore, the castor oil model may not be sensitive enough to evaluate the efficacy of treatments specifically targeting bacterial diarrhea, such as antibiotics or probiotics. The model’s response to treatments may not accurately reflect their effectiveness in a clinical setting where bacterial pathogens are involved.

### 3.2 Identification of compounds 1 and 2

**Figure 2:**
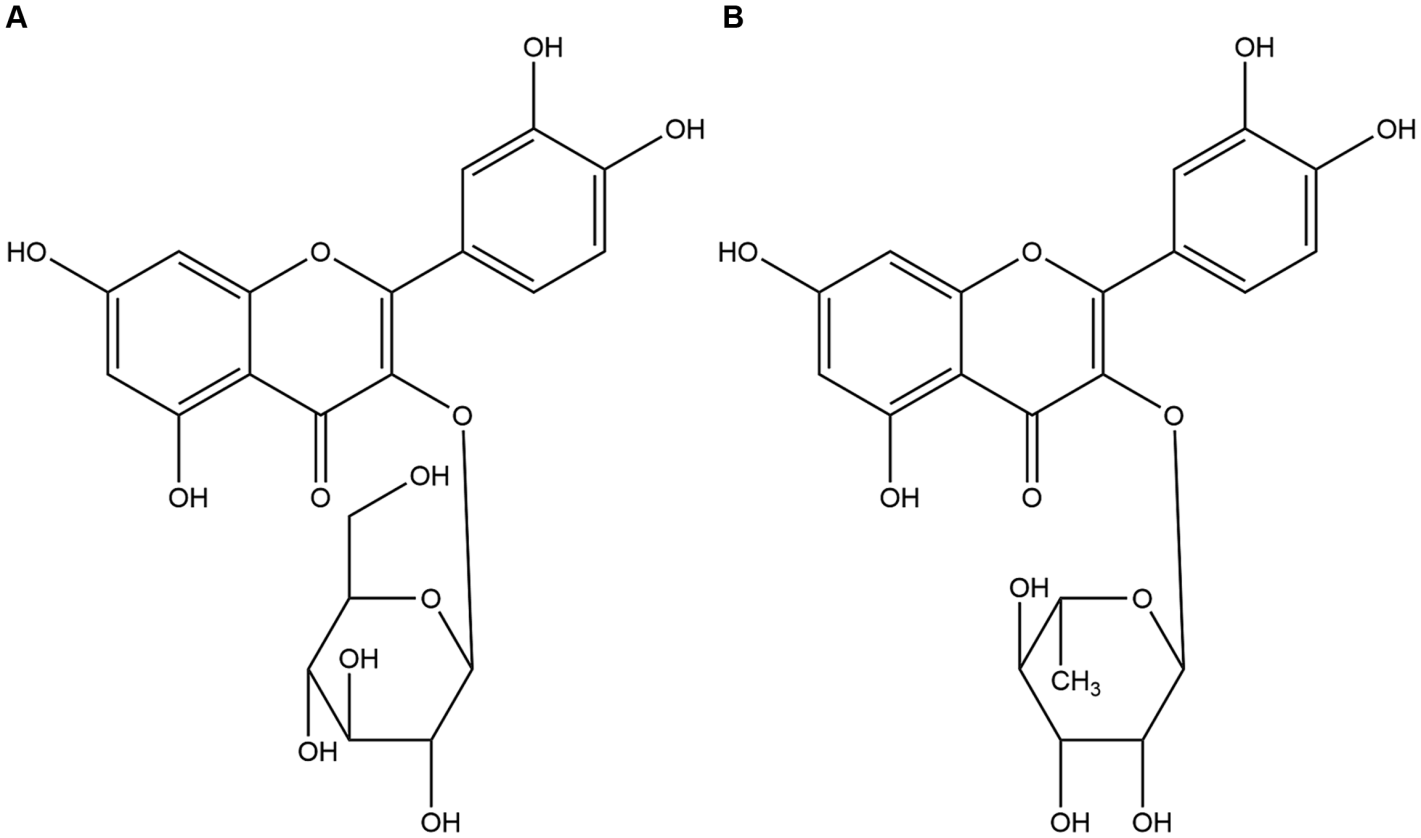
Chemical structures of isoquercitrin **(A)** and quercitrin **(B)**.

Utilizing a bioactivity-guided fractionation approach, the anti-diarrheal constituents were isolated. Compound 1 was characterized by HRESIMS, yielding a molecular formula of C_21_H_20_O_12_. The HRESIMS data revealed a sodium adduct ion at *m*/*z* 487.0831 [M + Na]^+^ (Calcd for C_21_H_20_O_12_Na, 487.3673) and a deprotonated molecular ion at *m*/*z* 463.0882 [M − H]^−^ (Calcd for C_21_H_19_O_12_, 463.3695). ^1^H NMR (600 MHz, DMSO-d_6_) δ: 12.660 (1H, s, 5-OH), 10.766 (1H, s, 7-OH), 9.556 (1H, s, 4′-OH), 9.256 (1H, s, 3′-OH), 7.310 (1H, d, *J* = 1.8 Hz, H-2′), 7.2625 (1H, dd, *J* = 8.4, 1.8 Hz, H-6′), 6.877 (1H, d, *J* = 8.4 Hz, H-5′), 6.3985 (1H, d, *J* = 1.8 Hz, H-8), 6.2125 (1H, d, *J* = 1.8 Hz, H-6), 5.242 (1H, d, *J* = 7.5 Hz, H-1″ of glucose), 3.807 to 3.407 (6H, m, H-2″-6″ of glucose). ^13^C-NMR (125 MHz, DMSO-d_6_) δ: 156.91 (C-2), 134.67 (C-3), 178.21 (C-4), 161.76 (C-5), 99.10 (C-6), 164.68 (C-7), 94.19 (C-8), 157.77 (C-9), 102.28 (C-10), 121.49 (C-1′), 116.04 (C-2′), 145.67 (C-3′), 148.90 (C-4′), 116.12 (C-5′), 121.34 (C-6′), 104.40 (C-1″), 71.66 (C-2″), 70.52 (C-3″), 70.82 (C-4″), 71.04 (C-5″), 62.66 (C-6″). By comparing these NMR data with literature values, compound 1 was conclusively identified as quercetin-3-O-β-D-glucopyranoside, commonly known as isoquercitrin (30), and its structure is depicted in Figure 2A.

Compound 2 was obtained as a white powder. HRESIMS data were instrumental in determining its molecular formula (C_21_H_20_O_11_). The observed sodium adduct ion at *m*/*z* 471.0945 [M + Na]^+^ (Calcd for C_21_H_20_O_11_Na, 471.3679) and the deprotonated molecular ion at *m*/*z* 447.0967 [M − H]^−^ (Calcd for C_21_H_19_O_11_, 447.3701). ^1^H NMR (600 MHz, DMSO-d_6_) δ: 12.660 (1H, s, 5-OH), 10.766 (1H, s, 7-OH), 9.556 (1H, s, 4′-OH), 9.256 (1H, s, 3′-OH), 7.310 (1H, d, *J* = 1.8 Hz, H-2′), 7.2625 (1H, dd, *J* = 8.4, 1.8 Hz, H-6′), 6.877 (1H, d, *J* = 8.4 Hz, H-5′), 6.3985 (1H, d, *J* = 1.8 Hz, H-8), 6.2125 (1H, d, *J* = 1.8 Hz, H-6), 0.826 (3H, d, *J* = 6.0 Hz, CH_3_ of rhamnose), 2.507–5.267 (8H, m, protons of rhamnose). ^13^C-NMR (100 MHz, DMSO-d_6_) δ: 156.91 (C-2), 134.67 (C-3), 178.21 (C-4), 161.76 (C-5), 99.10 (C-6), 164.68 (C-7), 94.19 (C-8), 157.77 (C-9), 102.28 (C-10), 121.49 (C-1′), 116.04 (C-2′), 145.67 (C-3′), 148.90 (C-4′), 116.12 (C-5′), 121.34 (C-6′), 104.40 (C-1″), 71.66 (C-2″), 70.52 (C-3″), 70.82 (C-4″), 71.04 (C-5″), 17.96 (C-6″). These data, when compared with those of compound 1, suggested that both compounds share the same flavonoid aglycone but differ in their glycosylation pattern. Specifically, the β-D-glucopyranoside found in compound 1 is replaced by an α-L-rhamnoside in compound 2, leading to the identification of compound 2 as quercetin-3-O-α-L-rhamnoside, commonly referred to as quercitrin, as depicted in Figure 2B.

### 3.3 Anti-diarrheal effects of isoquercitrin and quercitrin

#### 3.3.1 Effects of isoquercitrin and quercitrin on castor oil-induced diarrhea

As presented in Table 5, both isoquercitrin and quercitrin significantly reduced the quantity of wet fecal matter and delayed the onset of diarrhea in a dose-dependent manner (*p* < 0.05). Notably, isoquercitrin exhibited more potent antidiarrheal activity compared to quercitrin at equivalent doses. When compared with the standard anti-diarrheal medication, loperamide at a dosage of 2 mg kg^−1^, the highest dose of isoquercitrin demonstrated a comparable inhibitory effect on the diarrheal parameters. This finding is in line with the work of Gálvez et al. (25), who reported the beneficial effects of quercitrin in the management of chronic diarrhea. While quercitrin’s antidiarrheal potential has been previously acknowledged, this study marks the first instance of isoquercitrin being isolated from *E. hirta* and its antidiarrheal activity being experimentally validated.

**Table 5:** Effects of isoquercitrin and quercitrin on castor oil-induced diarrhea in mice.

Group	Dose (mg kg^−1^)	Mean of wet faeces	Onset of diarrhea (min)	Inhibition (%)
Negative control	10	10.2 ± 3.69	73.7 ± 2.49	—
Isoquercitrin	12.5	6.8 ± 3.56^*^	98.3 ± 5.32^**^	33.3
Isoquercitrin	25	5.6 ± 3.41^**^	106.5 ± 8.41^**^	45.1
Isoquercitrin	50	4.9 ± 3.35^**^	135.8 ± 7.41^**^	51.9
Quercitrin	12.5	7.4 ± 1.25^*^	87.1 ± 5.92^*^	27.5
Quercitrin	25	6.4 ± 1.37^**^	99.3 ± 3.67^**^	37.3
Quercitrin	50	5.2 ± 1.63^**^	123.2 ± 9.18^**^	49
Loperamide	2	4.8 ± 1.72^**^	157.4 ± 21.62^**^	52.9

Values were expressed as mean ± SEM (*n* = 6), ^*^p < 0.01 and ^**^p < 0.001 vs. negative control group.

Quercetin, one of the most potent antidiarrheal constituents derived from *E. hirta*, has been underutilized due to its limited oral absorption and bioavailability (31). However, the glucosylation of quercetin has been shown to enhance its water solubility, a modification that Morand et al. (32) confirmed to be advantageous for its absorption in the small intestine. Notably, the addition of a 3-O-rhamnose or 3-O-glucose-rhamnose moiety to the aglycone has been found to significantly reduce its absorption.

Our research corroborates these findings, demonstrating that isoquercitrin possesses a significant antidiarrheal effect, surpassing that of quercitrin at equivalent dosages (Table 5). This disparity in efficacy is likely due to the differential absorption profiles of isoquercitrin and quercitrin within the small intestine. Specifically, quercetin 3-glucose has been observed to be absorbed more efficiently in the small intestine compared to quercetin alone. It is plausible to deduce that the superior absorption efficiency of isoquercitrin is the primary factor contributing to its enhanced antidiarrheal activity over quercitrin.

#### 3.3.2 Effects of isoquercitrin and quercitrin on small intestinal propulsion

In contrast to the control group, both isoquercitrin and quercitrin elicited significant (*p* < 0.05) and dose-dependent decreases in normal intestinal transit across the dosage range of 12.5 to 50 mg kg^−1^, as detailed in Table 6. Notably, loperamide (2 mg kg^−1^) resulted in a 79.2% reduction in the small intestinal transit rate, a figure that closely mirrors the effect observed with the highest tested dose of isoquercitrin (50 mg kg^−1^). Furthermore, isoquercitrin demonstrated superior inhibitory effects on intestinal propulsion compared to an equivalent dose of quercitrin. These observed effects are hypothesized to substantially contribute to the antidiarrheal properties attributed to both isoquercitrin and quercitrin.

**Table 6:** Effects of isoquercitrin and quercitrin on gastrointestinal motility in mice.

Group	Dose (mg kg^−1^)	Propulsion length of activated charcoal (cm)	Propulsion inhibition (%)
Negative Control	10	11.1 ± 2.13	—
Isoquercitrin	12.5	5.83 ± 2.35^*^	47.5
Isoquercitrin	25	3.41 ± 2.86^**^	69.2
Isoquercitrin	50	2.50 ± 0.46^**^	77.5
Quercitrin	12.5	7.10 ± 1.32	36.0
Quercitrin	25	6.73 ± 0.68^*^	39.4
Quercitrin	50	4.80 ± 1.01^**^	56.8
Loperamide	2	2.31 ± 0.21^**^	79.2

Values were expressed as mean ± SEM (*n* = 5), ^*^p < 0.01 and ^**^p < 0.001 vs. negative control group.

#### 3.3.3 Effects of isoquercitrin and quercitrin on castor oil-induced enteropooling

Relative to the control group, pretreatment with isoquercitrin at dosages of 12.5, 25, and 50 mg kg^−1^, as well as quercitrin at equivalent dosages, resulted in a dose-dependent reduction in castor oil-induced fluid accumulation (Table 7). The positive control, loperamide at 2 mg kg^−1^, also exhibited a significant decrease in intestinal fluid accumulation, with an effect of 69.7%, which is nearly equivalent to that of the highest dose of isoquercitrin (50 mg kg^−1^), reflecting a 68.6% reduction. These findings suggest that the inhibition of intestinal fluid accumulation is a key mechanism underlying the antidiarrheal activity of both isoquercitrin and quercitrin.

**Table 7:** Effects of quercitrin and isoquercitrin on castor oil-induced enteropooling in rats.

Group	Dose (mg kg^−1^)	Volume of small intestinal effusion (v/mL)	Inhibition rate of small intestinal effusion (%)
Negative control	10	2.87 ± 0.66	—
Isoquercitrin	12.5	1.53 ± 0.84^*^	47.7
Isoquercitrin	25	1.33 ± 0.85^*^	53.7
Isoquercitrin	50	0.90 ± 0.57^**^	68.6
Quercitrin	12.5	2.10 ± 0.22	27.7
Quercitrin	25	2.05 ± 0.11	36.0
Quercitrin	50	1.73 ± 0.85	56.8
Loperamide	2	0.87 ± 0.17^**^	69.7

Values were expressed as mean ± SEM (*n* = 5), ^*^p < 0.05 and ^**^p < 0.01 vs. negative control group.

#### 3.3.4 Acute toxicity of isoquercitrin and quercitrin

Isoquercitrin and quercitrin exhibited no lethality or appreciable changes in behavior when administered at a dose of 5,000 mg kg^−1^ via a single oral dose, as observed over a 14-day period. This finding suggests that these two flavonoid glycosides possess a favorable safety profile, positioning them as potential lead compounds for the treatment of diarrhea. Notably, while the individual compounds identified in our study are non-toxic, the crude extract of *E. hirta* has shown toxicity in acute toxicity assays (33–35). Consequently, the use of the refined compounds, as opposed to the crude extract, is advisable to ensure safety.

### 3.4 Molecular docking

Castor oil is recognized for inducing diarrhea in mice, a phenomenon attributed to the release of ricinoleic acid through the hydrolysis of castor oil. This biochemical process precipitates a significant alteration in the transport of electrolytes and water, culminating in hypersecretion and pronounced contraction of intestinal smooth muscles (36). Furthermore, the endogenous opioid system (EOS) has been implicated in the pathophysiology of diarrhea, exerting its influence on gastrointestinal motility and the secretion/absorption dynamics of water and ions within the intestines. Among the constituents of the EOS, the delta-opioid receptor is notably prevalent in the intestine (37). Activation of this receptor by endogenous enkephalins is known to attenuate chloride ion and water secretion while simultaneously promoting fluid absorption in the intestinal lumen.

In the present study, loperamide is a synthetic piperidine moiety-based opioid served as the reference compound for assessing *in vivo* antidiarrheal activity. The active constituents isoquercitrin and quercitrin, isolated from *E. hirta*, exhibited antidiarrheal effects comparable to those of loperamide. To further elucidate the molecular basis of these effects, docking simulations were performed using the Autodock Vina software to evaluate the interactions of loperamide, isoquercitrin, and quercitrin with the delta-opioid receptor. The calculated affinities were −9.1, −8.5, and −8.2 kcal mol^−1^ for loperamide, isoquercitrin, and quercitrin, respectively, indicating that both flavonoid glycosides possess substantial binding affinity to the delta-opioid receptor, similar to loperamide. These findings suggest that isoquercitrin and quercitrin could be promising candidates for the treatment of secretory diarrhea, particularly if their bioavailability is optimized through innovative formulation strategies.

**Figure 3:**
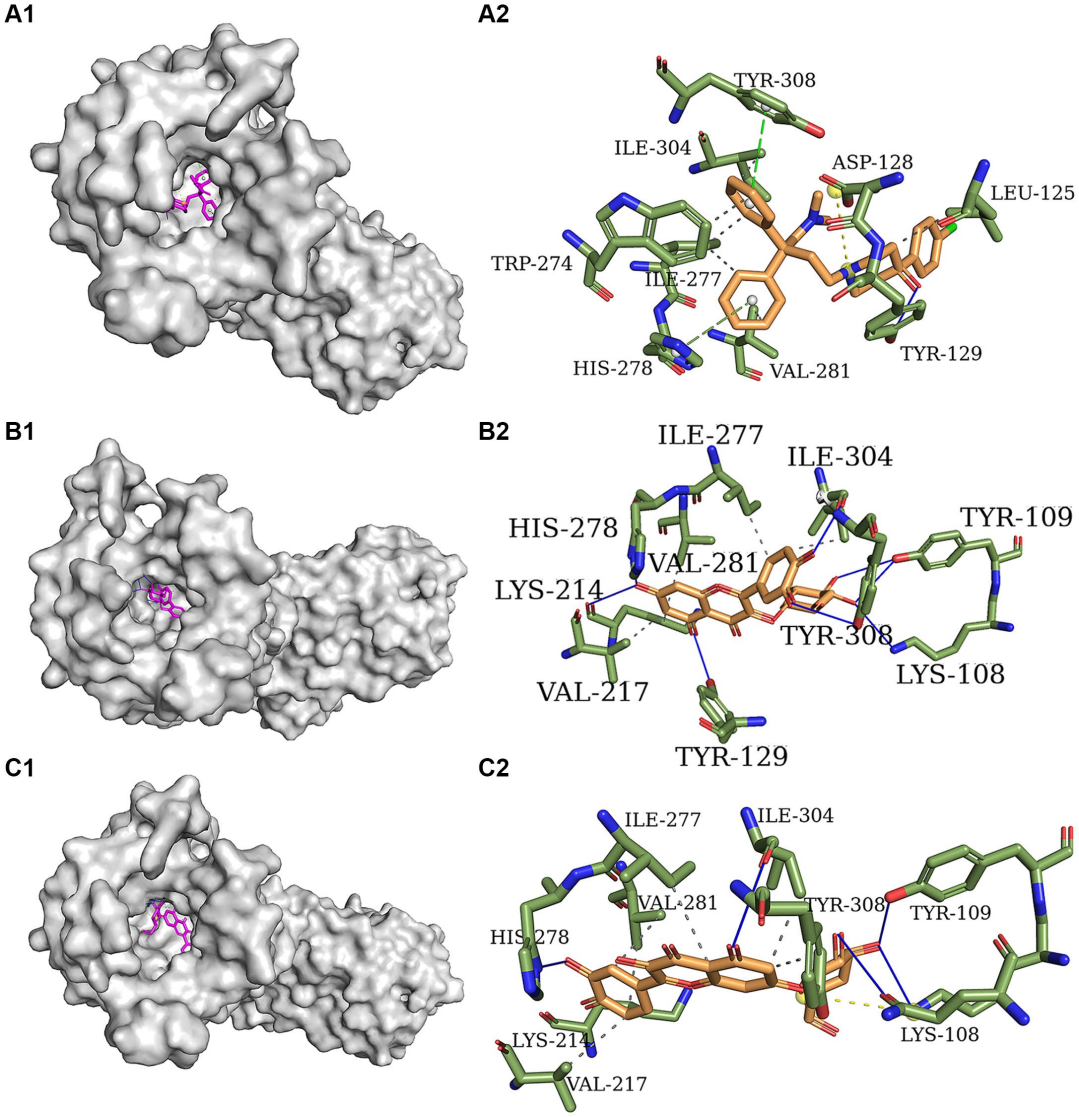
Molecular docking models of delta-opioid receptor and loperamide **(A)**, isoquercitrin **(B)**, and quercitrin **(C)**.

Figures 3A_1_–C_1_ depict the distinct yet overlapping binding of the three compounds within the delta-opioid receptor cavity. It is noteworthy that all ligands exhibit a similar pattern of engagement with the active binding site pocket. The docked orientations reveal a complex network of hydrophobic interactions, hydrogen bonds, *π*-stacking, and salt bridges between the ligands and the amino acids comprising the delta-opioid receptor. Specifically, Figure 3A_2_ illustrates that loperamide engages in hydrophobic interactions with Leu 125, Trp 274, Ile 277, Val 281, and Ile 304. Additionally, loperamide forms a strong hydrogen bond with Tyr 129, *π*-stacks with His 278 and Tyr 308, and establishes a salt bridge with Asp 128 within the delta-opioid receptor. Figures 3B_2_,C_2_ demonstrate that both isoquercitrin and quercitrin share hydrophobic interactions with Lys 214, Val 217, Ile 277, Val 281, and Ile 304, and form robust hydrogen bonds with Lys 108, Tyr 109, His 278, and Ile 304. Furthermore, isoquercitrin is distinguished by additional hydrogen bonds with Tyr 129, Lys 214, and Tyr 308, which may underlie the observed lower affinity in docking studies compared to quercitrin. The disparity in binding affinities among loperamide, isoquercitrin, and quercitrin can be attributed to the distinctive *π*-stacking interactions exhibited by loperamide.

### 3.5 Compound based pharmacokinetics and toxicity property analysis

According to the 5-rule of Lipinski, a compound is considered to have high oral bioavailability if it meets the five rules, such as molecular weight < 500 amu, hydrogen bond donor ≤5, hydrogen bond acceptor ≤10, LogP ≤5 and rotatable bonds ≤10. Table 8 illustrates that isoquercitrin and quercitrin do not fully comply with Lipinski’s parameters, suggesting that these two flavonoid glycosides exhibit relatively low oral bioavailability (HOB ranking: loperamide > isoquercitrin > quercitrin). This finding provides a logical explanation for the similar docking results of loperamide, isoquercitrin, and quercitrin with the delta-opioid receptor, despite the superior antidiarrheal efficacy of loperamide. Nevertheless, isoquercetin and quercetin demonstrate favorable intestinal absorption, a higher affinity for plasma protein binding, reduced potential for AMES mutagenicity, and a lower incidence of carcinogenic effects. These attributes suggest that these compounds are promising candidates for drug development. Improvements in their bioavailability through pharmaceutical technologies, such as solid dispersion, cyclodextrin inclusion complexes, and nanoparticle formulations, could significantly enhance their therapeutic potential.

**Table 8:** Absorption, digestion, metabolism, excretion, and toxicological (ADME/T) properties of the compounds for good oral bioavailability.

	Loperamide	Isoquercitrin	Quercitrin
PubChem CID	3,955	5,280,804	5,280,459
MW (g mol^−1^)	477.05	464.38	448.38
HBD	1	8	7
HBA	3	12	11
LogP (o/w)	5.09	−0.54	0.49
Rotatable bonds	7	4	3
HIA	0.9667	0.6468	0.7322
HOB	0.7857	0.7286	0.6857
PPB (100%)	0.85	0.797	1.002
CAR (binary)	0.8714	0.9857	0.9857
AM	0.7500	0.8100	0.7700
AOT log(1/(mol kg^−1^))	3.221	0.4045	0.5184

MW, molecular weight (acceptance range: <500); HBD, hydrogen bond donor (acceptance range: ≤5); HBA, hydrogen bond acceptor: (acceptance range: ≤10); LogP, high lipophilicity (acceptance range: ≤5); RB, rotatable bonds: (acceptance range: ≤10); HIA, human intestinal absorption probability; HOB, human oral bioavailability probability; PPB, plasma protein binding probability; CAR, carcinogenicity probability; AM, AMES mutagenesis probability, AOT, acute oral toxicity.

## 4 Conclusion

This study aimed to scientifically validate the traditional use of *E. hirta* as an antidiarrheal agent in folk medicine. The aqueous extract of *E. hirta* demonstrated a significant reduction in the quantity of wet fecal matter and a delayed onset of diarrhea in a dose-dependent manner (*p* < 0.05). Notably, the EF showed a significant therapeutic advantage over the control group in reducing the incidence of wet fecal matter and delaying the onset of diarrhea (*p* < 0.05, *p* < 0.01). Our investigation has clearly identified isoquercitrin and quercitrin as the principal bioactive constituents responsible for the observed antidiarrheal effects (*p* < 0.05). Molecular docking studies revealed that isoquercitrin and quercitrin possess substantial binding affinity to the delta-opioid receptor, with calculated affinities of −8.5 and −8.2 kcal mol^−1^, respectively, similar to loperamide at −9.1 kcal mol^−1^. However, the low oral bioavailability of isoquercitrin and quercitrin appears to constrain their therapeutic potential in the management of diarrhea. Consequently, further research is warranted to explore strategies for enhancing their bioavailability, thereby optimizing their anti-diarrheal efficacy.

## Data Availability

The structures of the delta opioid receptor in complex with loperamide, isoquercitrin, and quercitrin have been deposited in the National Microbiology Data Center (NMDC) https://nmdc.cn/resource/genomics/structure, accession number NMDCS0000141, NMDCS0000142 and NMDCS0000143.

## Footnotes

^1^ https://www.pymol.org/

^2^ https://plip-tool.biotec.tu-dresden.de/plip-web/plip/index

^3^ http://lmmd.ecust.edu.cn/admetsar2

## Glossary

HPLC: High-performance liquid chromatography
EF: Ethyl acetate fraction
BF: *n*-butanol fraction
AF: Aqueous fraction
ASF: Aqueous subfraction
ESF20: 20% ethanol subfraction
ESF30: 30% ethanol subfraction
ESF60: 60% ethanol subfraction
ESF90: 90% ethanol subfraction
HRESIMS: High-resolution electrospray ionization mass spectrometry
NMR: Nuclear magnetic resonance
CMC-Na: Sodium carboxymethyl cellulose
PDB: Protein Data Bank
RCSB: Research Collaboratory for Structural Bioinformatics
HBD: Human blood-brain barrier penetration
HBA: Human blood-albumin binding
LogP: Logarithm of the *n*-octanol/water partition coefficient
HIA: Human intestinal absorption
HOB: Hepatic organic anion binding
PPB: Plasma protein binding
ANOVA: Analysis of variance
EOS: Endogenous opioid system
