# Supplementary material for: Bioactivity-guided isolation of potential antidiarrheal constituents from Euphorbia hirta L. and molecular docking evaluation
Source: Front Vet Sci. 2024 Aug 29;11:1451615. doi: 10.3389/fvets.2024.1451615 (PMC11390441; doi:10.3389/fvets.2024.1451615)
Supplement: Supplementary file 1 [file Data_Sheet_1.docx]

**Supporting Information**

**Bioactivity-guided isolation of potential antidiarrheal constituents from *Euphorbia hirta* L. and molecular docking evaluation**

Junkai Wu^1^, Xiaomeng Zhang^2^, Liyang Guo^2^, Zunlai Sheng^2,3*^

^1^ School of Pharmacy, Quanzhou Medical College, Quanzhou 362011, China

^2^ College of Veterinary Medicine, Northeast Agricultural University, Harbin 150006, China

^3^ Heilongjiang Key Laboratory for Animal Disease Control and Pharmaceutical Development, Northeast Agricultural University, Harbin 150006, China

* Correspondence author: [shengzunlai@neau.edu.cn](mailto:shengzunlai@neau.edu.cn)

**Detailed information on the Hydrogen and Carbon Nuclear Magnetic Resonance spectra for isoquercitrin and quercitrin is presented from Figure S1 to Figure S4**

Figure S1: Hydrogen Nuclear Magnetic Resonance spectrum of isoquercitrin

Figure S2: Carbon Nuclear Magnetic Resonance spectrum of isoquercitrin

Figure S3: Hydrogen Nuclear Magnetic Resonance spectrum of quercitrin

Figure S4: Carbon Nuclear Magnetic Resonance spectrum of quercitrin

**Detailed information of the ESI-MS negative and positive ion scans for isoquercitrin and quercitrin is shown from Figure S5 to Figure S8**

Figure S5: ESI-MS negative ion scanning of isoquercitrin

Figure S6: ESI-MS positive ion scanning of isoquercitrin

Figure S7: ESI-MS negative ion scanning of quercitrin

Figure S8: ESI-MS positive ion scanning of quercitrin


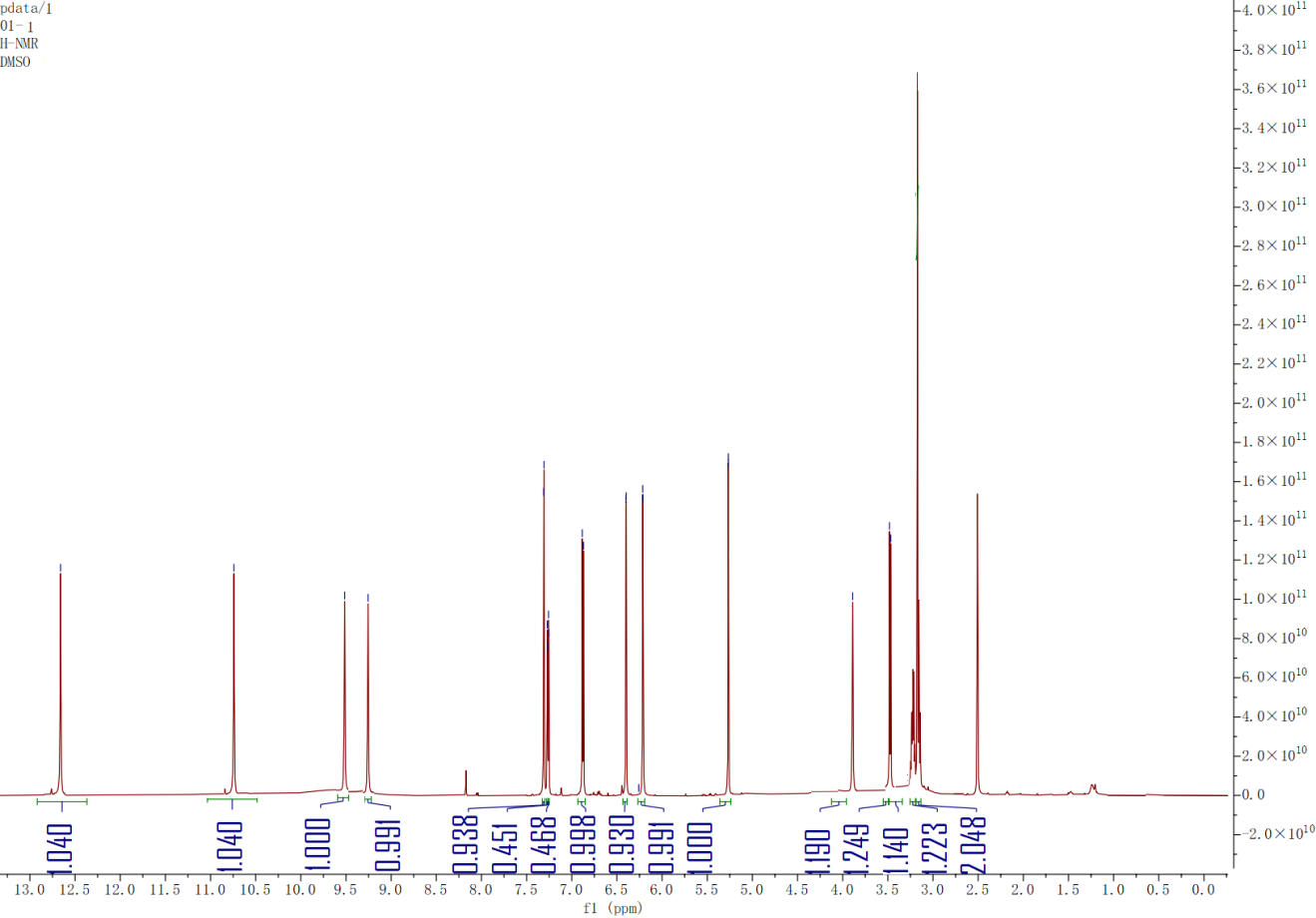


Figure S1 Hydrogen Nuclear Magnetic Resonance spectrum of isoquercitrin


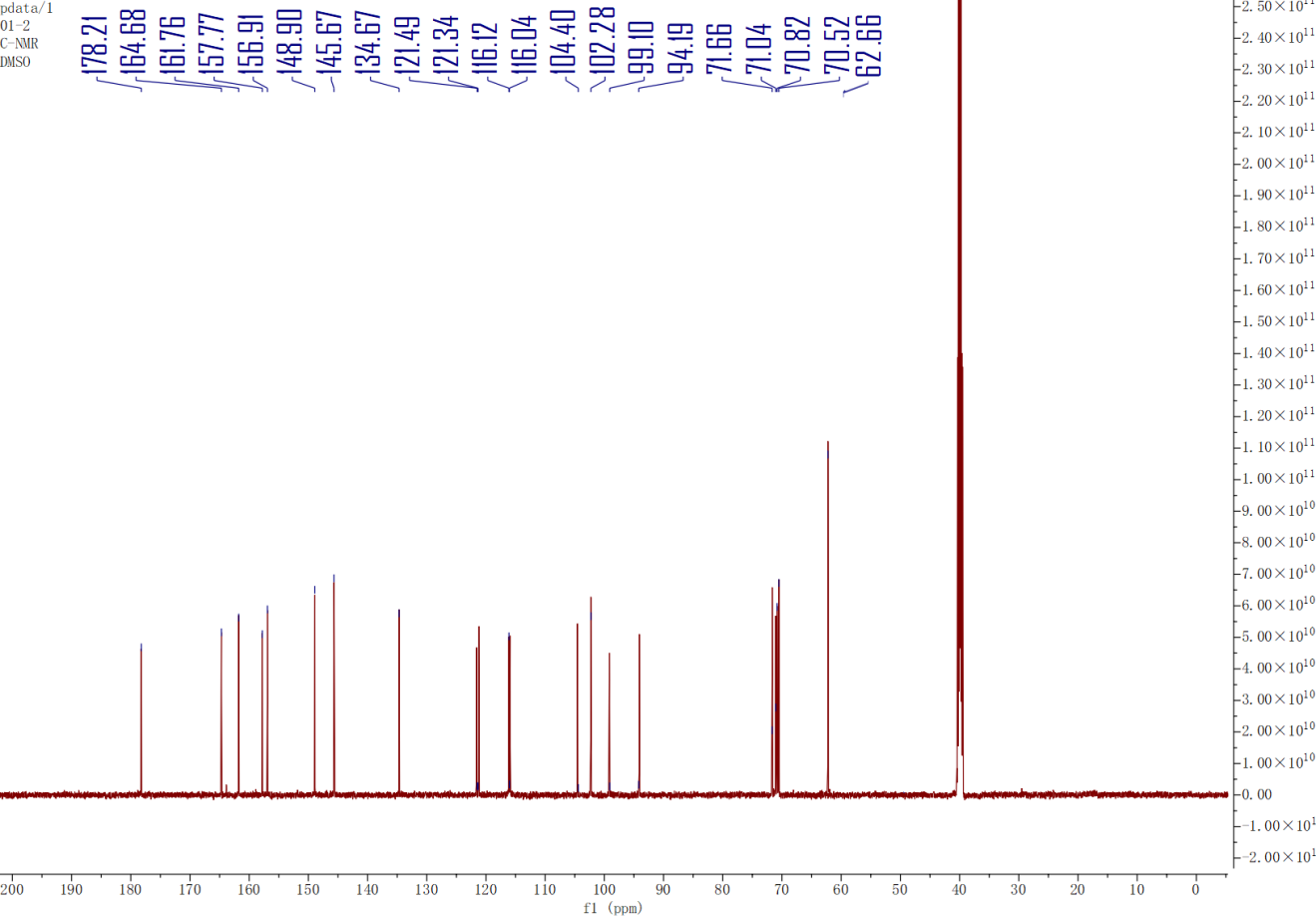


Figure S2 Carbon Nuclear Magnetic Resonance spectrum of isoquercitrin

**
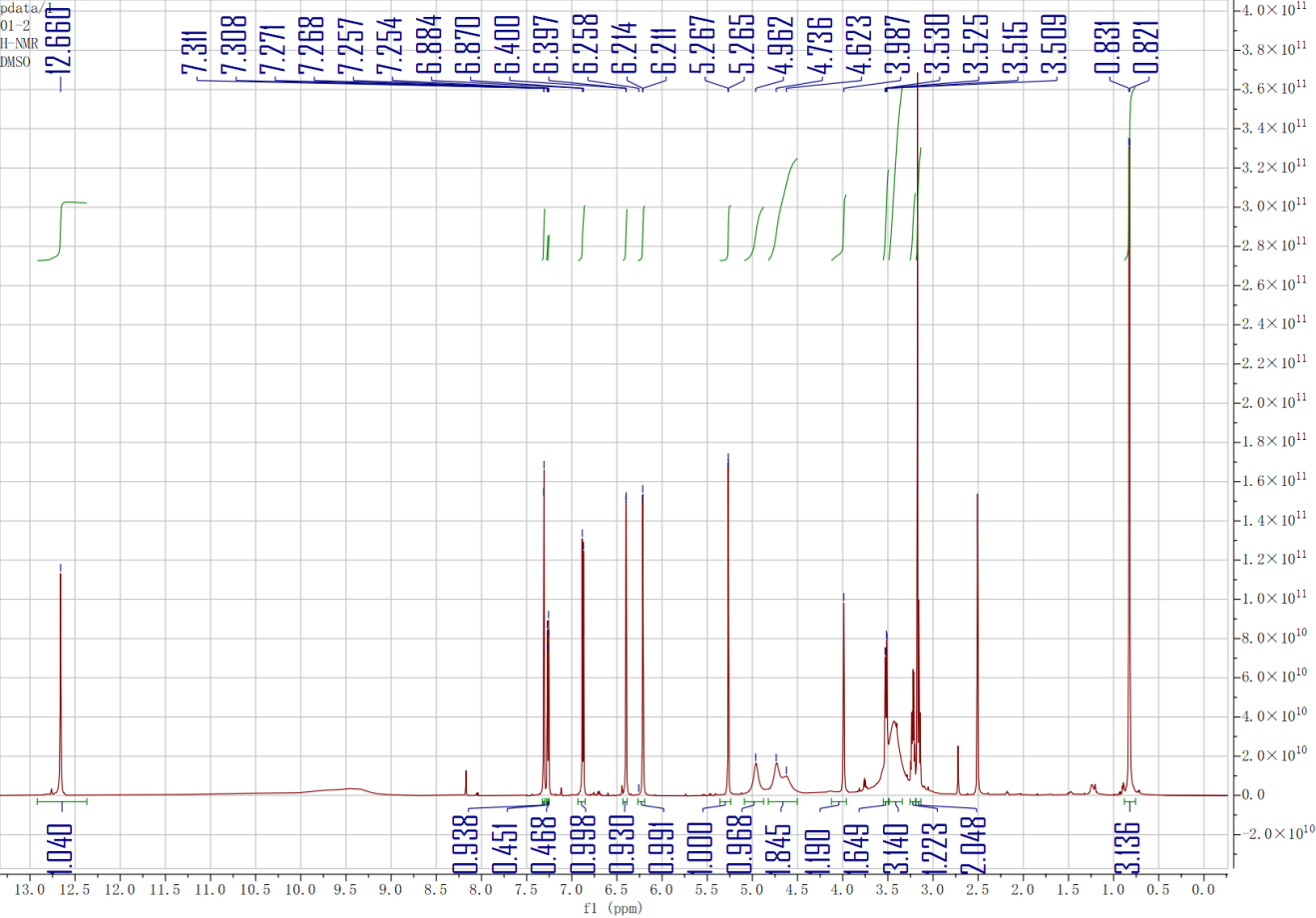
**

Figure S3 Hydrogen Nuclear Magnetic Resonance spectrum of quercitrin


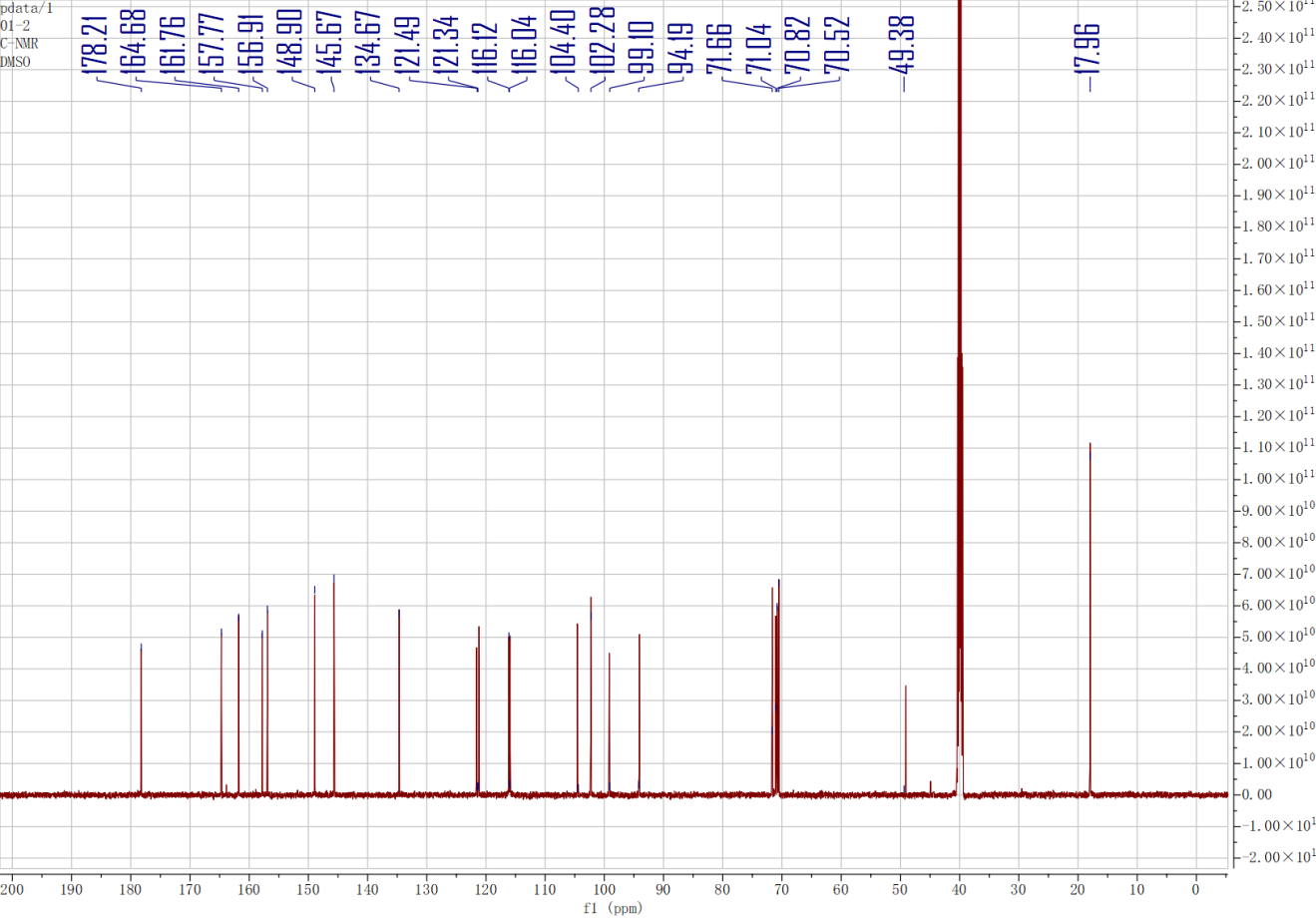


Figure S4 Carbon Nuclear Magnetic Resonance spectrum of quercitrin


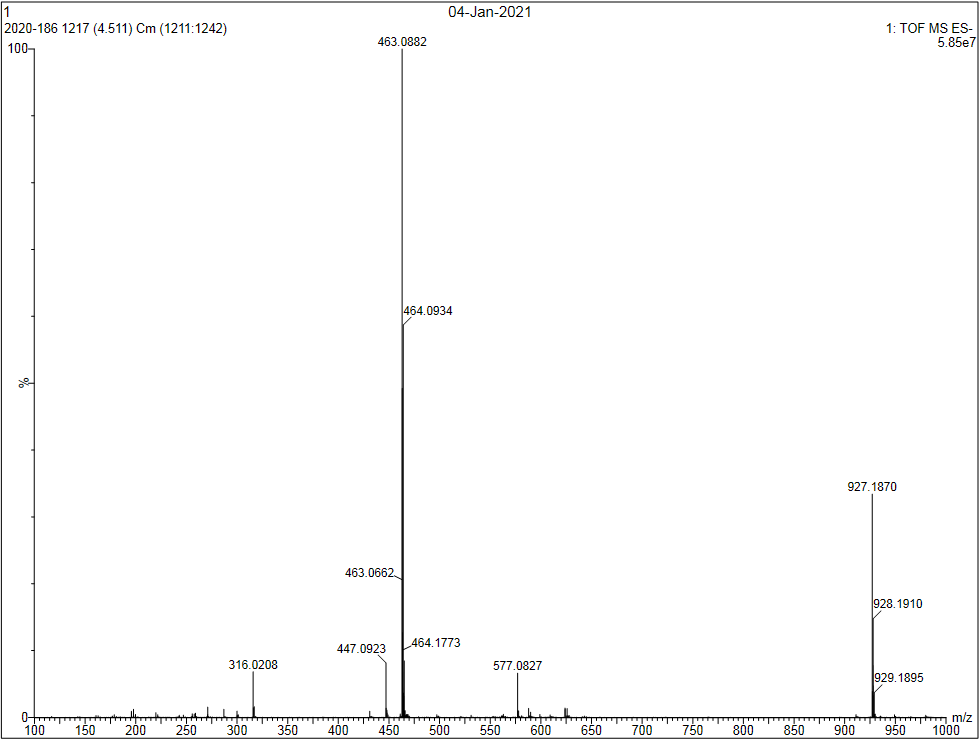


Figure S5 ESI-MS negative ion scanning of isoquercitrin


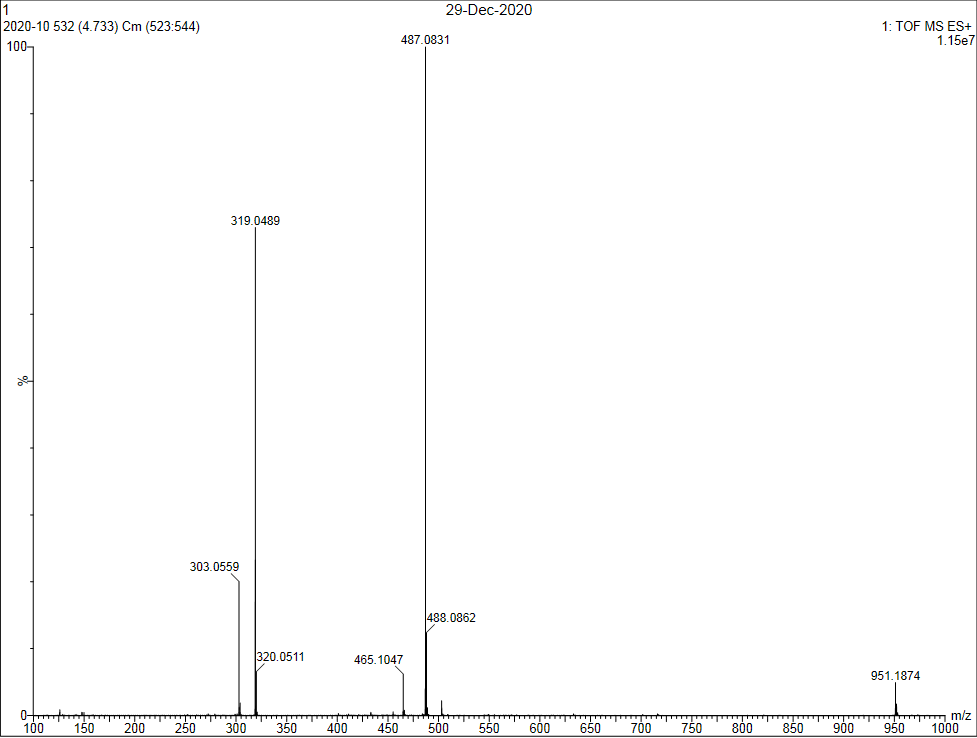


Figure S6 ESI-MS positive ion scanning of isoquercitrin


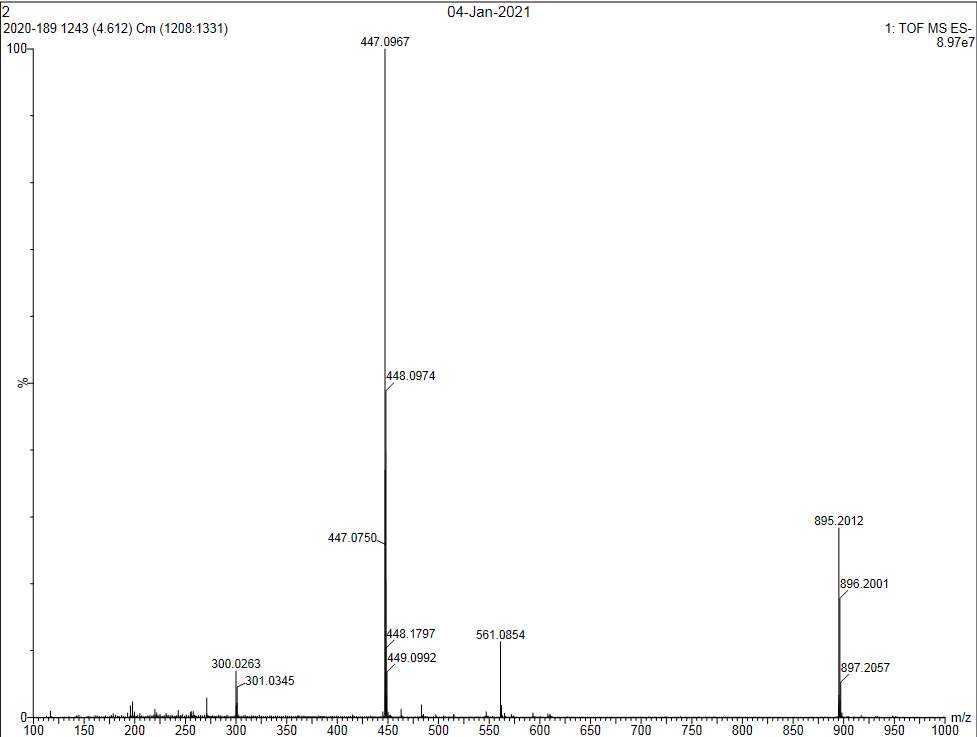


Figure S7 ESI-MS negative ion scanning of quercitrin


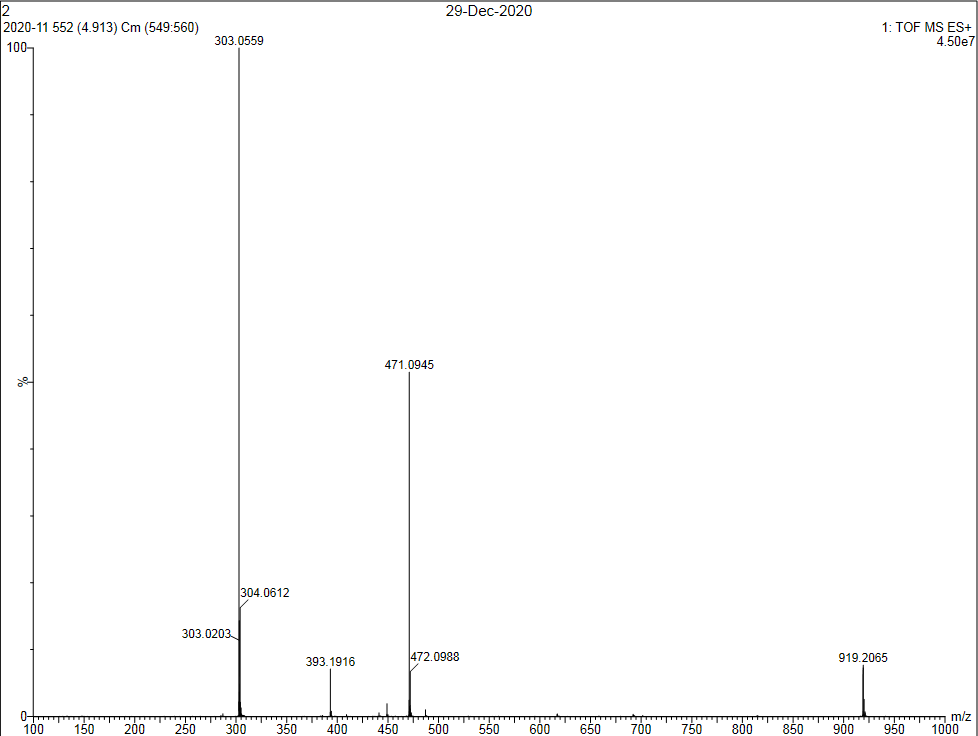


Figure S8 ESI-MS positive ion scanning of quercitrin
